# Ameliorating effect of chotosan and its active component, *Uncaria* hook, on lipopolysaccharide-induced anxiety-like behavior in mice

**DOI:** 10.3389/fphar.2024.1471602

**Published:** 2024-09-04

**Authors:** Yasumasa Okawa, Soichiro Ushio, Yasuhisa Izushi, Yoshihisa Kitamura, Yoshito Zamami, Toshiaki Sendo

**Affiliations:** ^1^ Department of Clinical Pharmacy, Graduate School of Medicine, Dentistry and Pharmaceutical Sciences, Okayama University, Okayama, Japan; ^2^ Department of Emergency and Disaster Medical Pharmacy, Faculty of Pharmaceutical Sciences, Fukuoka University, Fukuoka, Japan; ^3^ Department of Pharmacotherapy, School of Pharmacy, Shujitsu University, Okayama, Japan

**Keywords:** anxiolytic, chotosan, inflammation, serotonin receptor, *Uncaria* hook

## Abstract

**Introduction:**

In this study, we aimed to examine the effects of chotosan, a traditional Japanese botanical drug, and its active component, *Uncaria* hook, on anxiety-like behaviors induced by systemic inflammation in mice.

**Methods:**

To induce systemic inflammation, the mice were treated with lipopolysaccharide (LPS), a bacterial endotoxin. Prior to LPS treatment, the mice were administered chotosan or *Uncaria* hook orally each day for 14 days. Anxiety-like behavior of the mice was evaluated using the light–dark test 24 h after LPS treatment.

**Results:**

Repeated administration of chotosan prevented anxiety-like behavior in both normal and LPS-treated mice. Similarly, administration of *Uncaria* hook suppressed LPS-induced anxiety-like behavior in mice. Furthermore, treatment with tandospirone, a 5-HT_1A_ receptor agonist, alleviated anxiety-like behavior in mice, whereas treatment with DOI, a 5-HT_2A_ receptor agonist, enhanced anxiety-like behavior in mice. LPS treatment significantly increased serotonin (5-HT)_2A_ receptor mRNA expression in the frontal cortex, whereas 5-HT_1A_ receptor mRNA expression remained unchanged in the hippocampus. Notably, chotosan significantly suppressed the mRNA expression of 5-HT_2A_ receptor.

**Discussion:**

These findings indicate that chotosan exerts anxiolytic-like effects in the context of inflammation-induced anxiety, potentially mediated by the inhibition of 5-HT_2A_ receptor hyperfunction in LPS-treated mice. Consequently, we postulate that chotosan may be effective in managing inflammation-induced anxiety-like behaviors.

## 1 Introduction

Inflammation significantly contributes to psychiatric disorders, including anxiety, bipolar disorder, and depression (Leonard and Maes, 2012; Yang et al., 2015). Elevated levels of pro-inflammatory cytokines, such as interleukin-6 (IL-6), have been observed in patients with anxiety and major depressive disorders (Choi et al., 2022; Dowlati et al., 2010). Previous studies by our group and others have demonstrated that systemic administration of lipopolysaccharide (LPS), a bacterial endotoxin, elicits anxiety-like behavior in mice (Matsumoto et al., 2021; Sabedra Sousa et al., 2019; Ushio et al., 2022).

Anxiety disorders are characterized by excessive fear or worry, often accompanied by physiological symptoms such as increased heart rate, sweating, and trembling. Their known causes include genetic predisposition, environmental stressors, and neurochemical imbalances (Bandelow and Michaelis, 2015). Therapeutic strategies for anxiety include pharmacological treatments such as benzodiazepines and selective serotonin reuptake inhibitors, as well as non-pharmacological approaches such as cognitive-behavioral therapy (CBT) (Bandelow and Michaelis, 2015; Bandelow et al., 2017; Hofmann et al., 2012; Savignac et al., 2016).

Several subtypes of serotonin (5-HT) receptors, especially the 5-HT_2A_ receptor, are hypothesized to play a major role in the development of emotional memory as well as in mediating anxiety and defensive responses (Homberg, 2012; Murnane, 2019). It has been reported that LPS treatment markedly increases the expression of 5-HT_2A_ receptor proteins in the cortex of mice (Savignac et al., 2016). Upregulated expression of cortical 5-HT_2A_ receptors has been implicated in the central pathophysiological mechanisms of inflammation (Savignac et al., 2016). This pharmacological evidence supports the hypothesis that 5-HT_2A_ receptors modulate anxiety. Furthermore, numerous studies have suggested that 5-HT_1A_ receptors are involved in anxiety, depression, and their treatment (Kitamura et al., 2003; Kitamura and Nagatani, 1996). Tandospirone, an agonist of the 5-HT_1A_ receptor, is currently clinically used as an anxiolytic agent. However, the anxiolytic effects of 5-HT_1A_ receptor agonists in inflammatory conditions remain poorly understood.

Phytotherapy, the use of plant-based remedies, has been employed in traditional medicine systems worldwide. Notably, in Indian traditional medicine (ayurveda), *Withania somnifera* (ashwagandha) extracts have been used for their anxiolytic and anti-inflammatory properties. Studies have shown that ashwagandha can effectively reduce stress and anxiety levels in humans and animal models (Costa et al., 2023; Dar et al., 2015; Gupta and Kaur, 2018; Kaur et al., 2017; Maccioni et al., 2022; Pratte et al., 2014). Similarly, Kampo medicine, a traditional Japanese medicine, includes various phytotherapeutic remedies for anxiety and other conditions.

Chotosan, also known as Diago Teng San in Chinese and Jopungsan in Korean, is a Kampo formula composed of 10 botanical drugs and gypsum fibrosum. It is extensively used in clinical practice to treat brain and heart diseases (Satoh, 2013). Chotosan is typically prescribed for individuals with moderate physical strength who experience chronic conditions, such as headaches, dizziness, stiff shoulders, neurosis, and a predisposition to hypertension (Ministry of Health, Labour, and Welfare MHLW, 2021). In a previous study, chotosan ameliorated cognitive impairment and hippocampal neuronal loss in a common carotid artery occlusion model of vascular dementia (Jiang et al., 2019). In addition, chotosan has demonstrated antidepressant-like effects in mice (Sasaki-Hamada et al., 2017) and alleviated anxiety-like behavior in a mouse model of type 2 diabetes (Zhao et al., 2012). Thus, chotosan has demonstrated potential benefits for the treatment of psychiatric disorders. Moreover, Kampo medicines, which include crude drugs, such as *Uncaria* hook found in chotosan, are frequently prescribed for a broad spectrum of conditions ranging from mental disorders to physical weakness. *Uncaria* hook refers to the dried stem and hook of the *Uncaria* plant, which belongs to the family Rubiaceae (Ndagijimana et al., 2013; Liang et al., 2020). Although many botanical origins have been identified for *Uncaria* hook (Zhang et al., 2015), the Japanese Pharmacopoeia recognizes three primary sources: *Uncaria rhynchophylla* Miquel, *Uncaria sinensis* Haviland, and *Uncaria macrophylla* Wallich. Geissoschizine methyl ether, an indole alkaloid metabolite of *Uncaria* hook, reportedly alleviates aggressive behavior in socially isolated mice through a partial agonist effect on the 5-HT_1A_ receptor (Nishi et al., 2012).

In this study, we aimed to investigate the anxiolytic effects of chotosan and *Uncaria* hook on LPS-induced anxiety-like behavior in mice using a light–dark test. Additionally, we assessed the effects of chotosan on the mRNA expression of 5-HT_1A_ and 5-HT_2A_ receptors in LPS-treated mice.

## 2 Materials and methods

### 2.1 Animals

This study was conducted in compliance with the recommendations outlined in the Guide for Animal Experiments of the Advanced Science Research Center of Okayama University and Shujitsu University. The animal study protocol was approved by the Animal Care and Use Committee of the Advanced Science Research Center of Okayama University (OKU-2023527) and Shujitsu University (054–001). In total, 430 six-week-old male ICR mice were used in this study. The mice were purchased from Jackson Laboratory, Japan (Yokohama, Japan). The mice were housed in a climate-controlled room with a temperature of approximately 23°C ± 1°C and a humidity level of approximately 60%. They were housed in groups of five per cage under a regular light–dark cycle (lights on from 08:00 to 20:00 h). The mice were provided standard laboratory food and tap water.

### 2.2 Drugs

The following drugs were used in this study: LPS (from *Escherichia coli* O127:B8; Sigma-Aldrich, St. Louis, MO, United States), WAY100635 (Sigma-Aldrich), tandospirone (Sediel^®^ Tablets; Sumitomo Pharma, Osaka, Japan), and (±)-1-(2,5-dimethoxy-4-iodophenyl)-2-aminopropane (DOI; Sigma-Aldrich). The drugs were dissolved in saline solution and administered to the mice via intraperitoneal (i.p.) injection at a volume of 10 mL/kg. Chotosan (Lot No. 2190047010) and chotokou (*Uncaria* hook) (Lot. no.: 2201089010) were provided by Tsumura & Co., (Tokyo, Japan). Chotosan comprises 10 dried botanical drugs and gypsum fibrosum. Chotosan was used in the form of an extract powder made of the following raw materials: 3.0 parts Japanese Pharmacopoeia (JP) *Uncaria* Hook [*U. rhynchophylla* Miquel, *U. sinensis* Haviland, or *U. macrophylla* Wallich, hook], 3.0 parts JP *Citrus unshiu* peel [*C. unshiu* Marcowicz, or *Citrus reticulata* Blanco (*Rutaceae*), pericarpium], 2.0 parts JP *Chrysanthemum* flower [*Chrysanthemum indicum* Linné, or *Chrysanthemum morifolium* Ramatulle, capitulum], 2.0 parts JP *Saposhnikovia* root and rhizome [*Saposhnikovia divaricata* Schischkin, root, and rhizome], 3.0 parts JP *Pinellia* tuber [*Pinellia ternata* Breitenbach, tuber], 3.0 parts JP *Ophiopogon* root [ *Opiopogon japonicus* Ker-Gawlerm, root], 3.0 parts JP *Poria sclerotium* [*Wolfiporia cocos* Ryvarden et Gilbertson, sclerotium], 2.0 parts JP Ginseng [*Panax ginseng* C. A. Meyer (*Panax schinseng Nees*) (*Araliaceae*), radix], 1.0 part JP Ginger [*Zingiber officinale* Roscoe (*Zingiberaceae*), rhizoma], 1.0 part JP Glycyrrhiza [*Glycyrrhiza uralensis* Fischer, or *Glycyrrhiza glabra* Linné (*Leguminosae*), radix], and 5.0 parts JP gypsum fibrosum [CaSO_4_ 2H_2_O]. The plants were identified based on their morphology and marker components, as per JP and company standards. The extract quality was standardized based on good manufacturing practices, as defined by the Ministry of Health, Labour, and Welfare of Japan. The concentrate was spray-dried to obtain the chotosan powder. A three-dimensional high-performance liquid chromatogram of chotosan, provided by Tsumura & Co., is shown in Supplementary Figure 1. The chotosan and *Uncaria* hook powders were dissolved in distilled water and administered by peroral (p.o.) injection.

### 2.3 Light–dark test

The light–dark test serves as an anxiogenic challenge, as it assesses the conflict between the desire to explore new environments and aversion to brightly lit zones (Ushio et al., 2022). We evaluated the preventative effects of repeated administration of chotosan and *Uncaria* hook solutions and tandospirone on LPS-induced anxiety-like behavior in mice using the light–dark test. The light–dark box consisted of a light zone (20 cm × 20 cm × 25 cm) and a dark zone (20 cm × 20 cm × 25 cm) with black walls and a black floor. The two zones were separated by a partition with a single opening (5 cm × 8 cm) to allow the mice to move between them. The light zone was illuminated at an intensity of 500 × l, whereas the dark zone was covered with a lid to prevent illumination. Individual mice were placed in the box for a total duration of 10 min. At the beginning of the test, the mice were placed in the center of the light zone, and the total time spent in the light zone was recorded. The floor of the light–dark box was covered with breeding wooden chips. These chips were replaced for each experiment. In addition, the behavior of the mice was recorded on video, and one researcher measured the time spent by mice in the light zone.

### 2.4 Locomotor activity

Locomotor activity was monitored for 10 min using automated activity monitoring chambers (DAS-8; Neuroscience, Inc., Tokyo, Japan). The plastic chambers measured 28 cm (width) × 20 cm (length) × 13 cm (height). Locomotor activity of mice was recorded for 10 min. The assay was conducted the day after the final administration of chotosan and *Uncaria* hook. Different mice were used for the locomotor activity test and the light–dark box test.

### 2.5 *In vivo* experimental schedule

Mice were injected with LPS (500 μg/kg, i. p.) 1 day prior to the light–dark test. The injection schedule and doses of LPS (500 μg/kg, i. p.) selected were based on our previous studies (Matsumoto et al., 2021; Ushio et al., 2022). Chotosan (100–1,000 mg/kg) or *Uncaria* hook (10–100 mg/kg) were administered orally (p.o.) once a day for 14 days until the day before the experiments. The doses and duration of the injection of chotosan and *Uncaria* hook were based on a previous report (Nishi et al., 2012). Following the administration of the final dose of chotosan or *Uncaria* hook, the animals were injected with LPS after a duration of 1 h. Single doses of tandospirone (0.1–1 mg/kg, i. p.) and DOI (1 mg/kg, i. p.) were administered intraperitoneally 30 min before the light–dark test. The doses of tandospirone and DOI were based on our previous study (Nakamura et al., 2018). Previous studies, including our own, have shown that tandospirone exhibits significant anxiolytic effects following a single administration in animal models (Nakamura et al., 2018; Shimizu et al., 1992). In the antagonism experiment using WAY100635, we performed the light–dark test at 1 h after the final administration of chotosan or *Uncaria* hook. WAY100635, a 5-HT_1A_ receptor antagonist, was administered 15 min before the light–dark test. The dose of WAY100635 was based on a previous study (Egashira et al., 2008). Mice used for the light–dark test and those used for the locomotor activity assay were different, because we considered that anxiety induced by the light–dark test could influence the locomotor activity.

### 2.6 Measurement of the frontal cortex 5-HT_2A_ receptor mRNA and hippocampal 5-HT_1A_ receptor mRNA expression using real-time quantitative polymerase chain reaction

At 24 h after LPS treatment, the mice were decapitated without anesthesia. The hippocampus and frontal cortex were collected, quickly frozen, and stored at −80°C until homogenization. Total RNA was isolated and purified from individual hippocampus and frontal cortex tissues using the Maxwell^®^ RSC RNA tissue kit (Promega, Madison, WI, United States), according to the manufacturer’s instructions. Thereafter, 1 µg of total RNA was reverse-transcribed to cDNA using ReverTra Ace^®^ quantitative reverse transcription polymerase chain reaction (RT-qPCR) master mix (TOYOBO, Osaka, Japan). Quantitative real-time PCR was performed using a StepOnePlus Real-Time PCR system (Thermo Fisher Scientific, Waltham, MA, United States) and THUNDERBIRD^®^ SYBR^®^ qPCR mix (TOYOBO) under the following amplification conditions: 95°C for 20 s for early denaturation, followed by 40 cycles for 1 s at 95°C for heat denaturation and 20 s at 60°C for annealing. To quantify the total expression of *Htr1a* and *Htr2a*, forward and reverse primers and probes were designed (Integrated DNA Technologies, Coralville, IO, United States). *Gapdh* was used as an internal control. The relative fold changes in the expression level of each target gene were calculated using the comparative CT method and the 2^−ΔΔCT^ equation. The sequences of the primers used, target genes, and other related information are presented in Table 1.

**TABLE 1:** Oligonucleotide sequences of the primer sets for RT-PCR.

Gene	Accession number	Forward (5′to 3′)	Reverse (5′to 3′)	Amplicon (bp)	Position (5′–3′)
*Htr1a*	NM_008308.5	Ctg​tga​cct​gtt​tat​cgc​cct​g	Gta​gtc​tat​agg​gtc​ggt​gat​tgc	109	1,046–1,154
*Htr2a*	NM_172812.3	Tca​cca​ttg​cgg​gaa​aca​t	Atc​agc​tat​ggc​aag​tga​cat	101	1,319–1,419
*Gapdh*	NM_008084.4	Ctc​tgc​tcc​tcc​ctg​ttc​ta	Gat​acg​gcc​aaa​tcc​gtt​cac	105	4–108

RT-PCR, reverse transcription polymerase chain reaction.

### 2.7 Statistical analysis

Data are presented as mean ± standard error of the mean. Data analysis was conducted using the Shapiro–Wilk test to assess the normality of data distribution before selecting the appropriate statistical tests. For normally distributed data, the student’s *t*-test or one-way or two-way analysis of variance (ANOVA) was used, followed by Tukey’s test (Excel-Toukei ver 7.0; ESUMI Co., Ltd., Tokyo, Japan) to determine the significance of differences among the groups. Statistical significance was set at *p* < 0.05. In cases where the number of animals was reduced, this occurred solely due to administration errors that resulted in the death of the animals, as detailed in the Methods section. No data were excluded based on statistical outliers or significant deviations from other data points.

## 3 Results

### 3.1 Effect of chotosan and *Uncaria* hook on LPS-induced anxiety-like behavior during the light–dark test in mice

**FIGURE 1:**
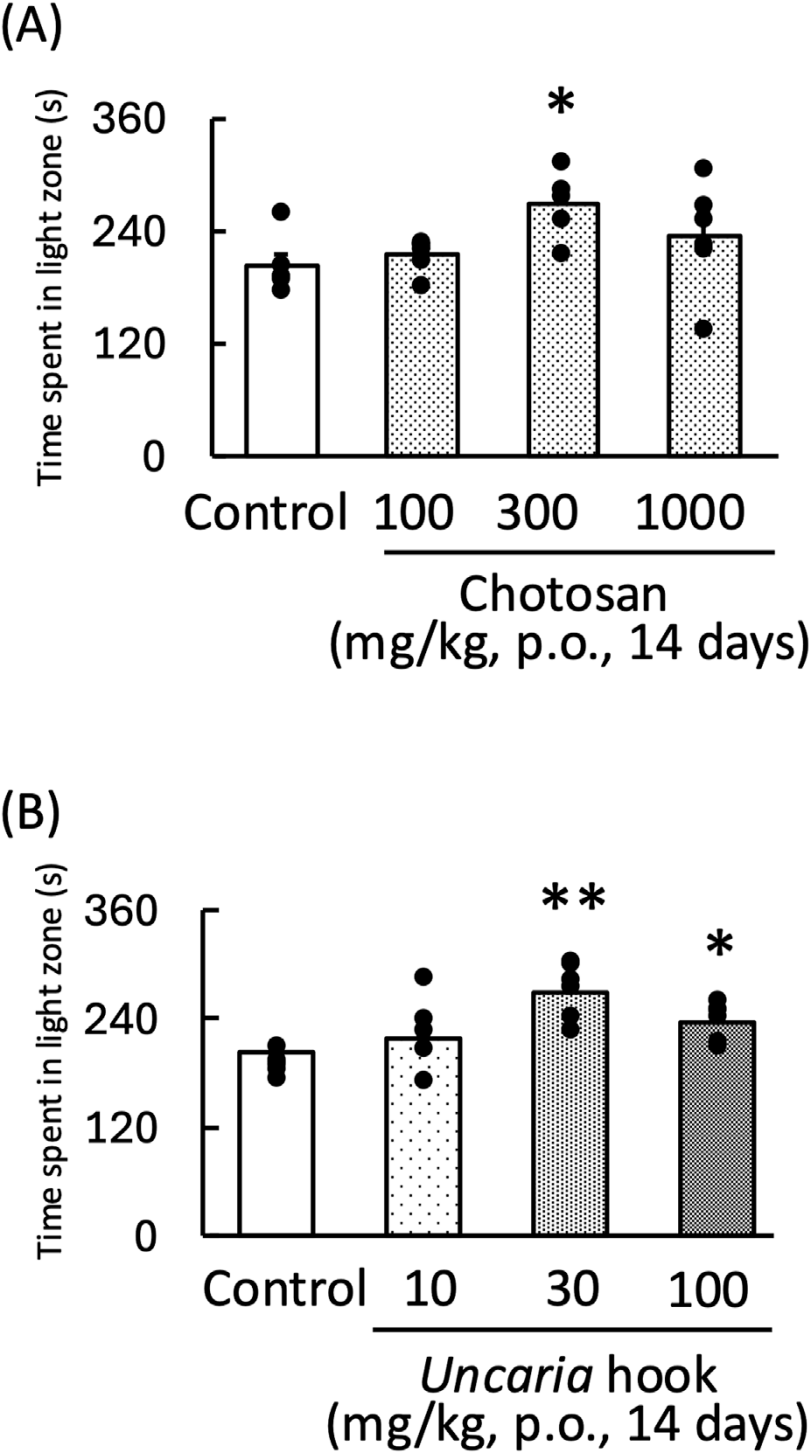
Effects of chotosan **(A)** and *Uncaria* hook **(B)** on the amount of time mice spent in the light zone during the light–dark test. Chotosan (100, 300, and 1,000 mg/kg, p. o.) or *Uncaria* hook (10, 30, and 100 mg/kg, p. o.) was administered for 14 days prior to the light–dark test. Data are shown as mean ± standard error of the mean; n = 5–6 per group. Data were analyzed using one-way ANOVA, and Tukey’s test was used to compare the means of multiple groups. *p < 0.05 (vs. control), **p < 0.01 (vs. control).

**FIGURE 2:**
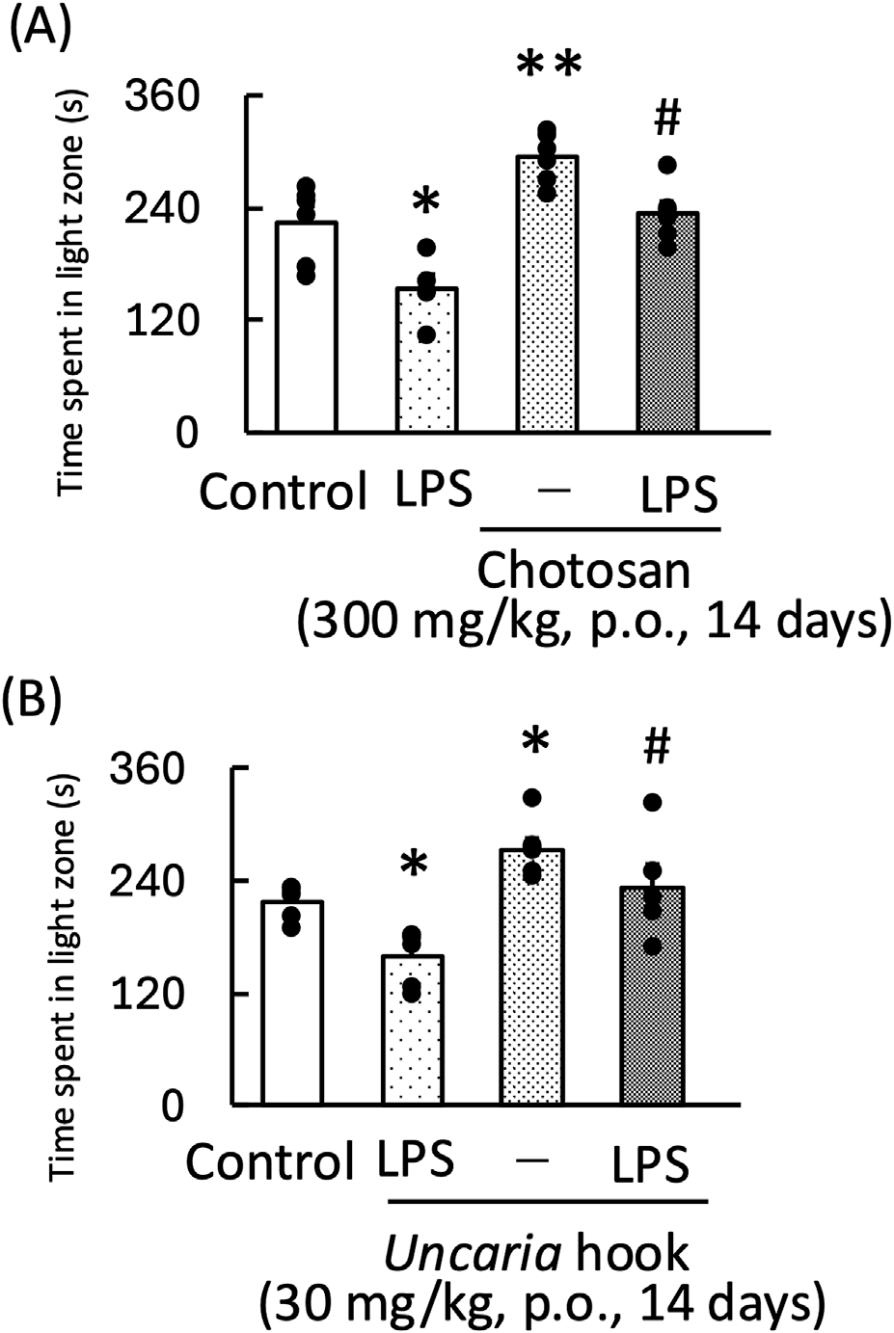
Effects of chotosan **(A)** and *Uncaria* hook **(B)** on the amount of time LPS-treated mice spent in the light zone during the light–dark test. Chotosan (300 mg/kg, p. o.) or *Uncaria* hook (30 mg/kg, p. o.) was administered for 14 days prior to the light–dark test. LPS (500 μg/kg, i.p.) was administered 1 day prior to the light–dark test. Data are shown as mean ± standard error of the mean; n = 6 per group. Data were analyzed using two-way ANOVA, and Tukey’s test was used to compare the means of multiple groups. *p < 0.05 (vs. control), **p < 0.01 (vs. control), #*p* < 0.05 (vs. LPS) i.p., intraperitoneal; LPS, lipopolysaccharide.

Treatment with both chotosan (300–1,000 mg/kg) and *Uncaria* hook (30–100 mg/kg) significantly increased the amount of time the mice spent in the light zone [chotosan: F (3,19) = 3.60, *p* < 0.05; *Uncaria* hook: F (3,19) = 8.65, *p* < 0.01] (Figures 1A, B). In contrast, LPS treatment significantly reduced the amount of time the mice spent in the light zone. In the LPS-treated mice, treatment with effective dose of both chotosan (300 mg/kg) and *Uncaria* hook (30 mg/kg) significantly increased the amount of time the mice spent in the light zone [chotosan: LPS: F (1, 11) = 10.48, *p* < 0.01; chotosan: F (1, 11) = 12.99, *p* < 0.01; LPS × chotosan F (1, 11) = 1.10, *p* = 0.32] (Figure 2A) [*Uncaria* hook: LPS: F (1, 11) = 17.63, *p* < 0.01; *Uncaria* hook: F (1, 11) = 38.96, *p* < 0.01; LPS × *Uncaria* hook: F (1, 11) = 0.48, *p* = 0.51] (Figure 2B). In the course of the experiment, we observed the death of two mice due to administration errors. Specifically, in Figure 1A, 1 mouse in the 300 mg/kg chotosan group died on day 3, and in Figure 1B, 1 mouse in the 10 mg/kg *Uncaria* hook group died on day 10. We believe that the cause of death was likely due to the administered substance inadvertently entering the trachea instead of the esophagus, leading to respiratory failure, as the deaths occurred immediately after oral administration.

### 3.2 Effect of chotosan and *Uncaria* hook on the locomotor activity in mice

**FIGURE 3:**
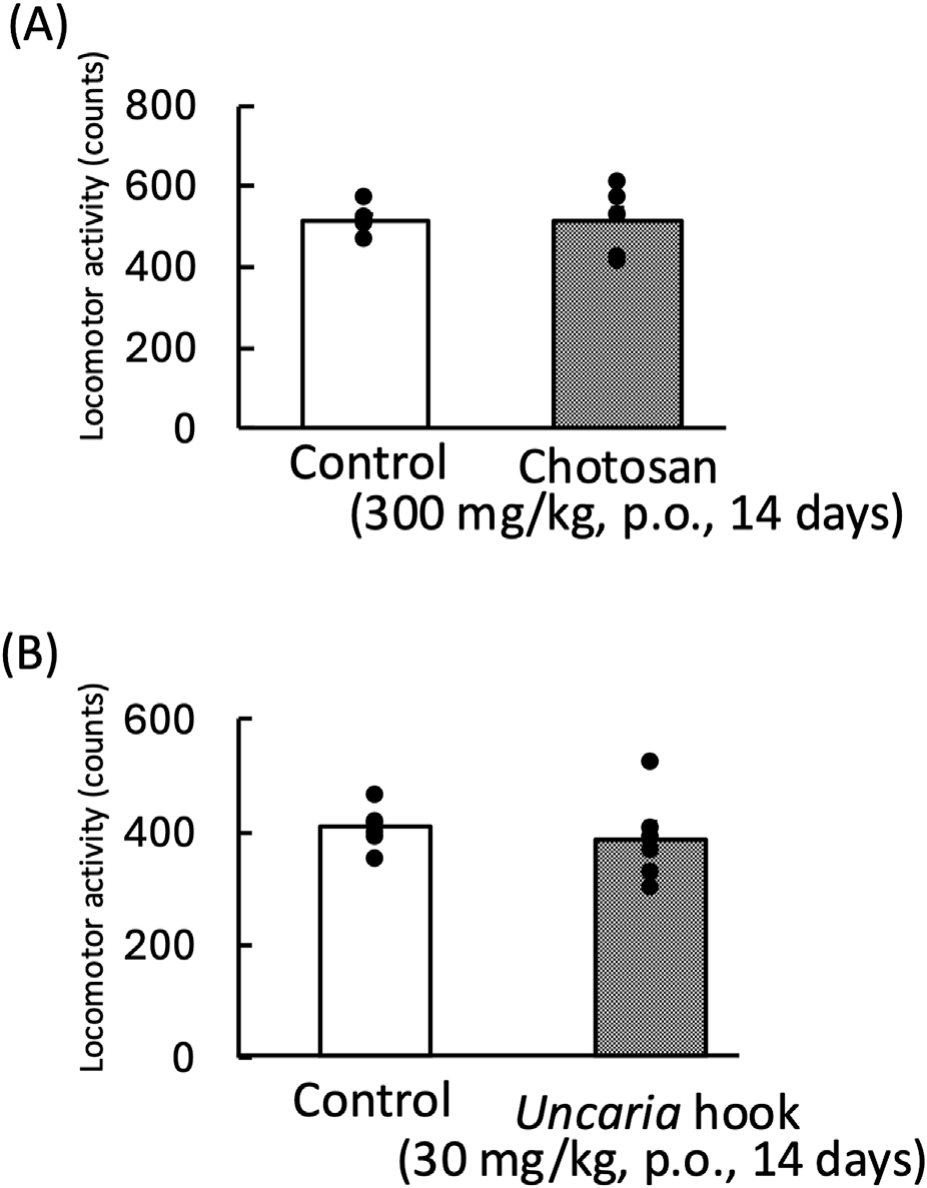
Effects of chotosan **(A)** and *Uncaria* hook **(B)** on locomotor activity in mice. Chotosan (300 mg/kg) or *Uncaria* hook (30 mg/kg) was administered orally once a day for 14 days until the day prior to locomotor activity measurement. The locomotor activity was monitored for 10 min. Data are shown as mean ± standard errors of the mean; n = 6 per group. Data were analyzed using the student’s *t*-test.

We evaluated the effect of chotosan (300 mg/kg) and *Uncaria* hook (30 mg/kg) on the locomotor activity of mice. Chotosan and *Uncaria* hook did not change the locomotor activity (chotosan: *p* = 0.95; *Uncaria* hook: *p* = 0.58) (Figure 3).

### 3.3 Effect of tandospirone on LPS-induced anxiety-like behavior during the light–dark test and locomotor activity in mice

**FIGURE 4:**
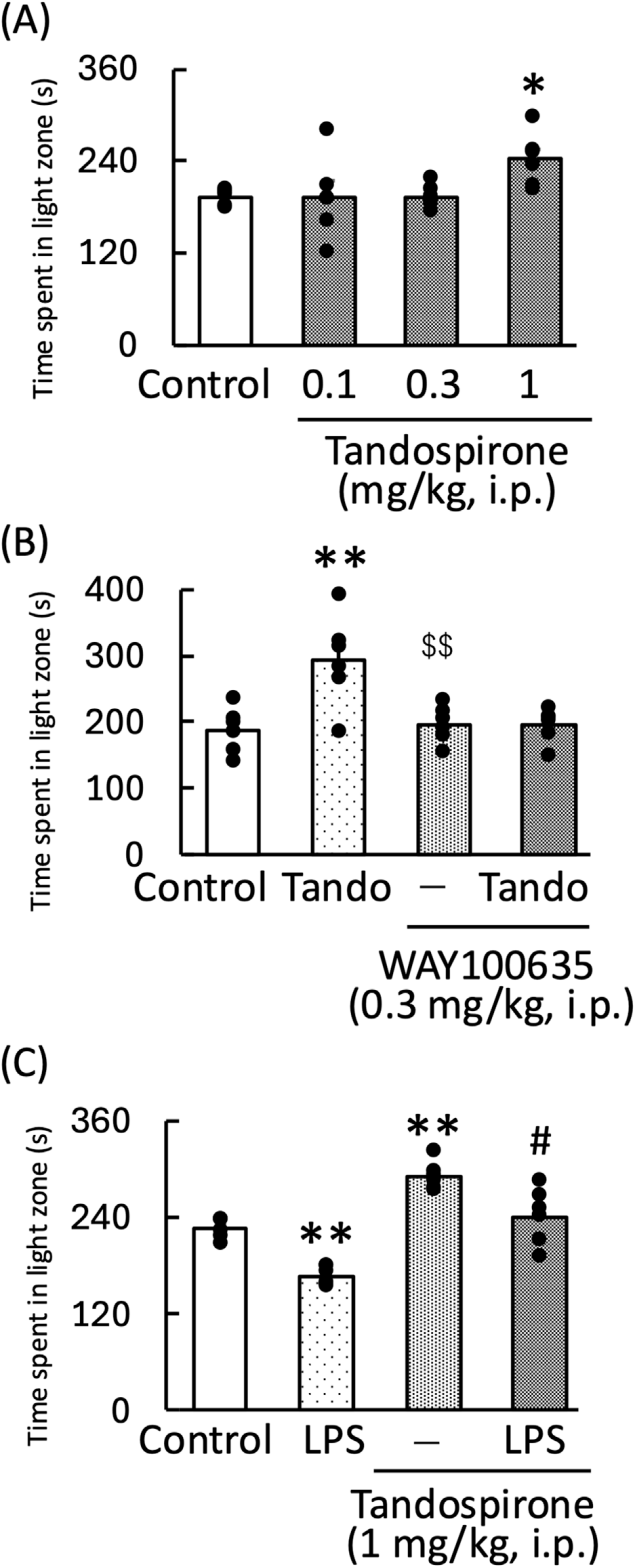
Effects of tandospirone on the amount of time spent by the normal **(A,B)** and LPS-treated **(C)** mice in the light zone during the light–dark test. Tandospirone (0.1, 0.3, and 1 mg/kg, i. p.) **(A)** and tandospirone (1 mg/kg, i. p.) **(B,C)** were administered 30 min before the light–dark test. WAY100635 (0.3 mg/kg, i. p.) was administered to normal mice 15 min before the light–dark test. LPS (500 μg/kg, i. p.) was administered 1 day prior to the light–dark test. Data are shown as mean ± standard error of the mean; n = 6 per group. Data were analyzed using one-way ANOVA; group means were compared using two-way ANOVA, and Tukey’s test was used to compare the means of multiple groups. *p < 0.05 (vs. control), **p < 0.01 (vs. control), $$*p* < 0.01 (vs. tandospirone), #*p* < 0.05 (vs. LPS). i.p., intraperitoneal; LPS, lipopolysaccharide; Tando, tandospirone.

**FIGURE 5:**
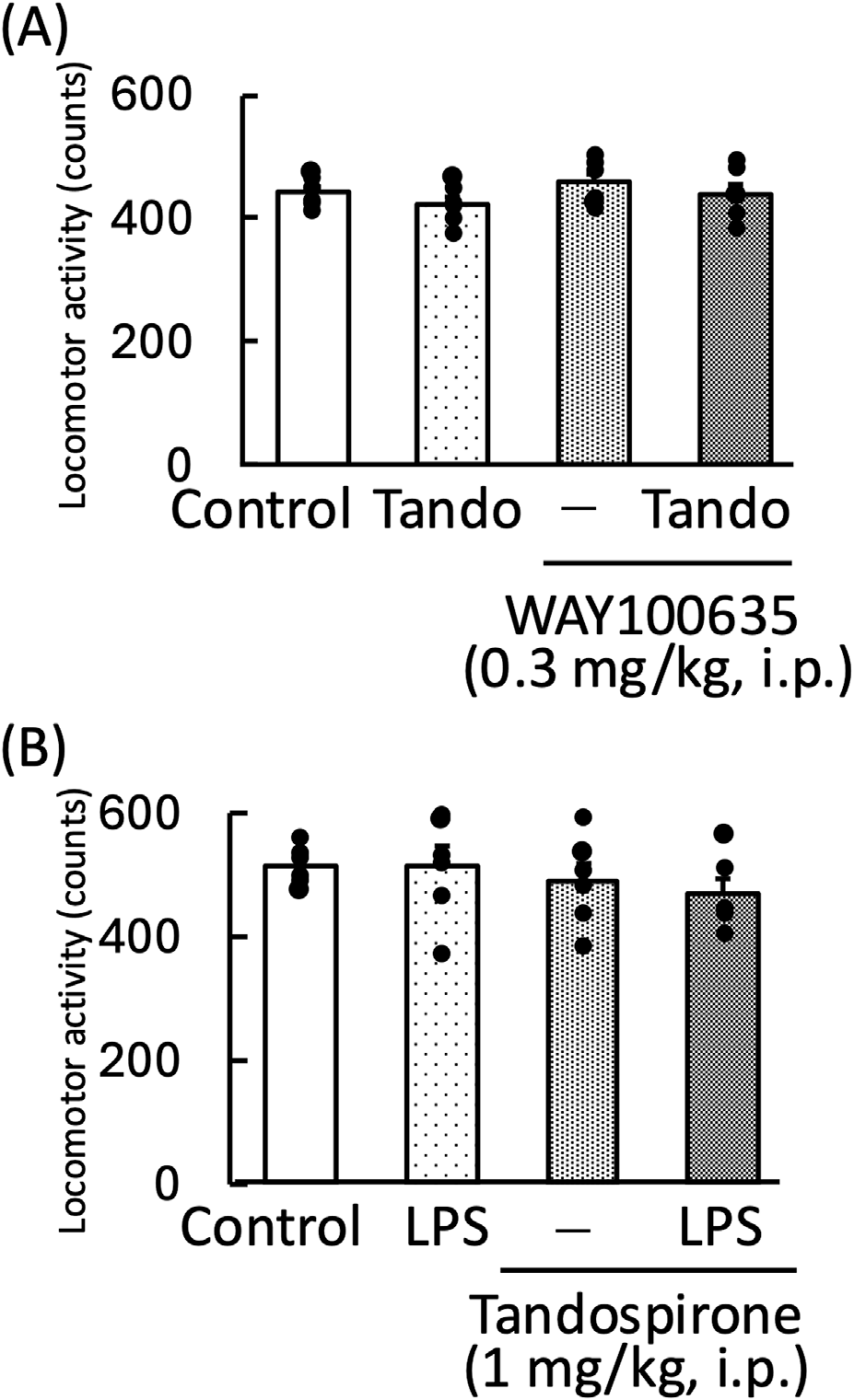
Effects of tandospirone on locomotor activity in WAY100635-treated **(A)** and LPS-treated **(B)** mice. Tandospirone (1 mg/kg, i. p.) was administered 30 min before the locomotor activity measurement. WAY100635 (0.3 mg/kg, i. p.) was administered 15 min before the light–dark test. LPS (500 μg/kg, i. p.) was administered 1 day prior to the locomotor activity measurement. The locomotor activity was monitored for 10 min. Data are shown as mean ± standard error of the mean; n = 6 per group. Data were analyzed using one-way ANOVA, group means were compared using two-way ANOVA, and Tukey’s test was used to compare the means of multiple groups. i.p., intraperitoneal; LPS, lipopolysaccharide; Tando, tandospirone.

Tandospirone, a 5-HT_1A_ receptor agonist, significantly increased the amount of time the mice spent in the light zone [F (3,20) = 3.35, *p* < 0.05] (Figure 4A). The anxiolytic effect of tandospirone was blocked by WAY100635, a 5-HT_1A_ receptor antagonist [WAY100635: F (1, 11) = 4.09, *p* = 0.07; tandospirone: F (1, 11) = 4.46, *p* = 0.06; WAY100635 × tandospirone: F (1, 11) = 4.70, *p* = 0.05] (Figure 4B). Additionally, tandospirone significantly increased the amount of time the LPS-treated mice spent in the light zone [LPS: F (1, 11) = 63.60, *p* < 0.01; tandospirone: F (1, 11) = 61.94, *p* < 0.01; LPS × tandospirone: F (1, 11) = 0.07, *p* = 0.80] (Figure 4C). However, the measurement of locomotor activity in this treatment group showed no significant changes [tandospirone: F (1, 11) = 7.73, *p* < 0.05; WAY100635: F (1, 11) = 0.84, *p* = 0.38; tandospirone × WAY100635: F (1, 11) = 0.002, *p* = 0.96] (Figure 5A); [LPS: F (1, 11) = 0.27, *p* = 0.62; tandospirone: F (1, 11) = 2.24, *p* = 0.16; LPS × tandospirone: F (1, 11) = 0.17, *p* = 0.68] (Figure 5B).

### 3.4 Effect of WAY100635 on the anxiolytic effects of chotosan and *Uncaria* hook on mice during the light–dark test

**FIGURE 6:**
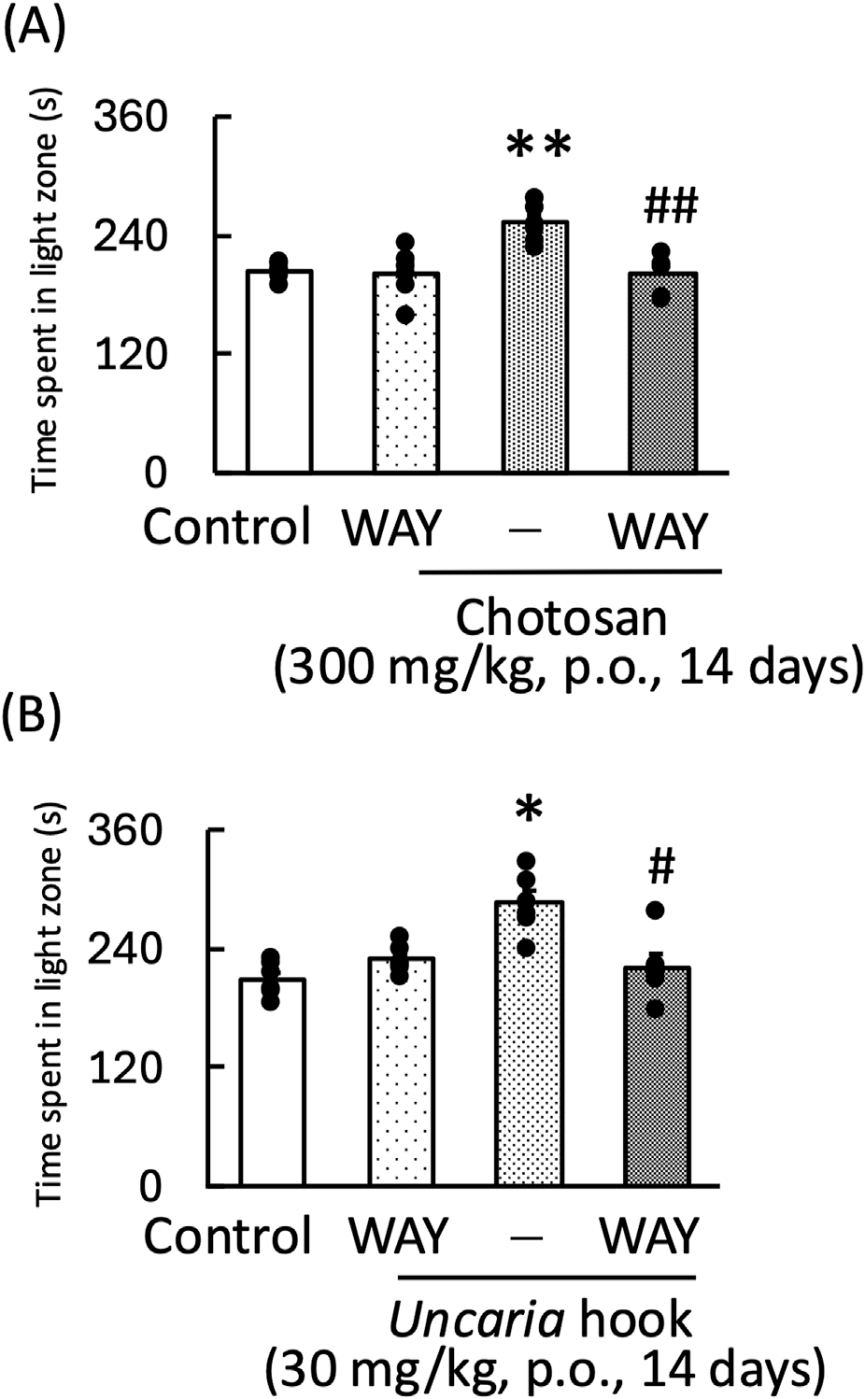
Effects of WAY100635 on chotosan-induced **(A)** and *Uncaria* hook-induced **(B)** increase in the amount of time spent by mice in the light zone during the light–dark test. Chotosan (300 mg/kg, p. o.) or *Uncaria* hook (30 mg/kg, p. o.) was administered for 14 days prior to the light–dark test. The light–dark test performed 1 h after the final administration of chotosan and *Uncaria* hook. WAY100635 (0.3 mg/kg, i. p.) was administered 15 min before the light–dark test. Data are shown as mean ± standard error of the mean; n = 6 per group. Data were analyzed using two-way ANOVA, and Tukey’s test was used to compare the means of multiple groups. *p < 0.05 (vs. control), **p < 0.01 (vs. control), #*p* < 0.05 (vs. chotosan), ##*p* < 0.01 (vs. *Uncaria* hook) i.p., intraperitoneal; p.o., peroral.

In the light–dark test, the anxiolytic effects of chotosan and *Uncaria* hook on mice were blocked by WAY100635, a 5-HT_1A_ receptor antagonist [WAY100635: F (1, 11) = 6.14, *p* < 0.05; chotosan: F (1, 11) = 4.36, *p* = 0.06; WAY100635 × chotosan: F (1, 11) = 4.38, *p* = 0.06] (Figure 6A) [WAY100635: F (1, 11) = 2.24, *p* = 0.16; *Uncaria* hook: F (1, 11) = 0.87, *p* = 0.37; WAY100635 × *Uncaria* hook: F (1, 11) = 16.22, *p* < 0.05] (Figure 6B).

### 3.5 Effect of DOI on LPS-induced anxiety-like behavior during the light–dark test and locomotor activity in mice

**FIGURE 7:**
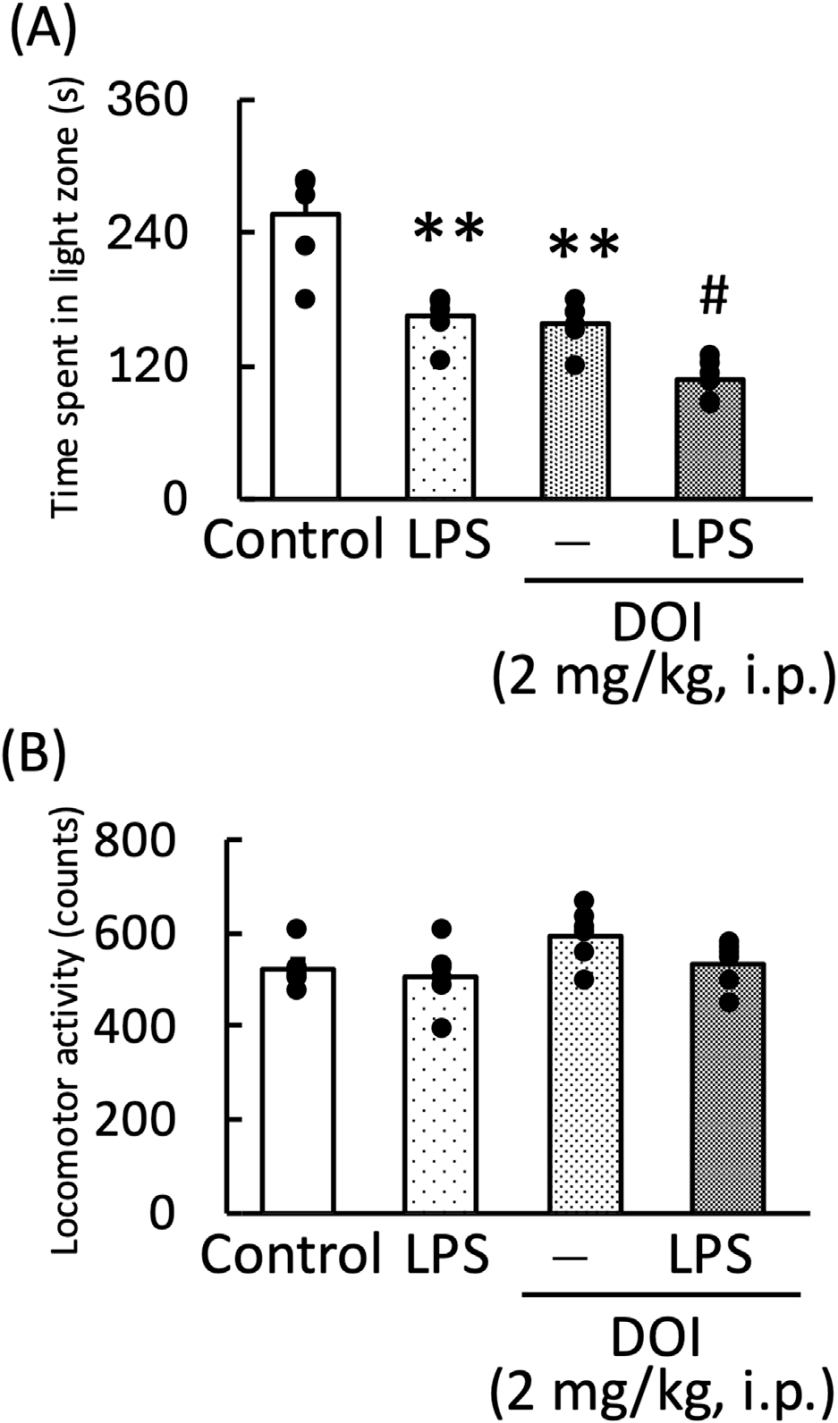
Effects of DOI on the amount of time mice spent in the light zone during the light–dark test **(A)** and locomotor activity of mice **(B)**. DOI (1 mg/kg, i. p.) was administered 30 min before the light–dark test and locomotor activity measurement. LPS (500 μg/kg, i. p.) was administered 1 day prior to the light–dark test and locomotor activity measurement. Data are shown as mean ± standard error of the mean; n = 6 per group. Data were analyzed using two-way ANOVA, and Tukey’s test was used to compare the means of multiple groups. **p < 0.01 (vs. Control), #*p* < 0.05 (vs. DOI) i.p., intraperitoneal.

DOI, a 5-HT_2A_ receptor agonist, significantly decreased the amount of time spent in the light zone by both normal and LPS-treated mice [LPS: F (1, 11) = 33.96, *p* < 0.01; DOI: F (1, 11) = 32.70, *p* < 0.01; LPS × DOI: F (1, 11) = 0.73, *p* = 0.73] (Figure 7A). However, the measurement of locomotor activity in this treatment group showed no significant changes [LPS: F (1, 11) = 3.32, *p* = 0.10; DOI: F (1, 11) = 11.59, *p* < 0.01; LPS × DOI: F (1, 11) = 0.87, *p* = 0.37] (Figure 7B).

### 3.6 Effects of chotosan and *Uncaria* hook on the mRNA expression of frontal cortex 5-HT_2A_ receptor and hippocampal 5-HT_1A_ receptor in LPS-treated mice

**FIGURE 8:**
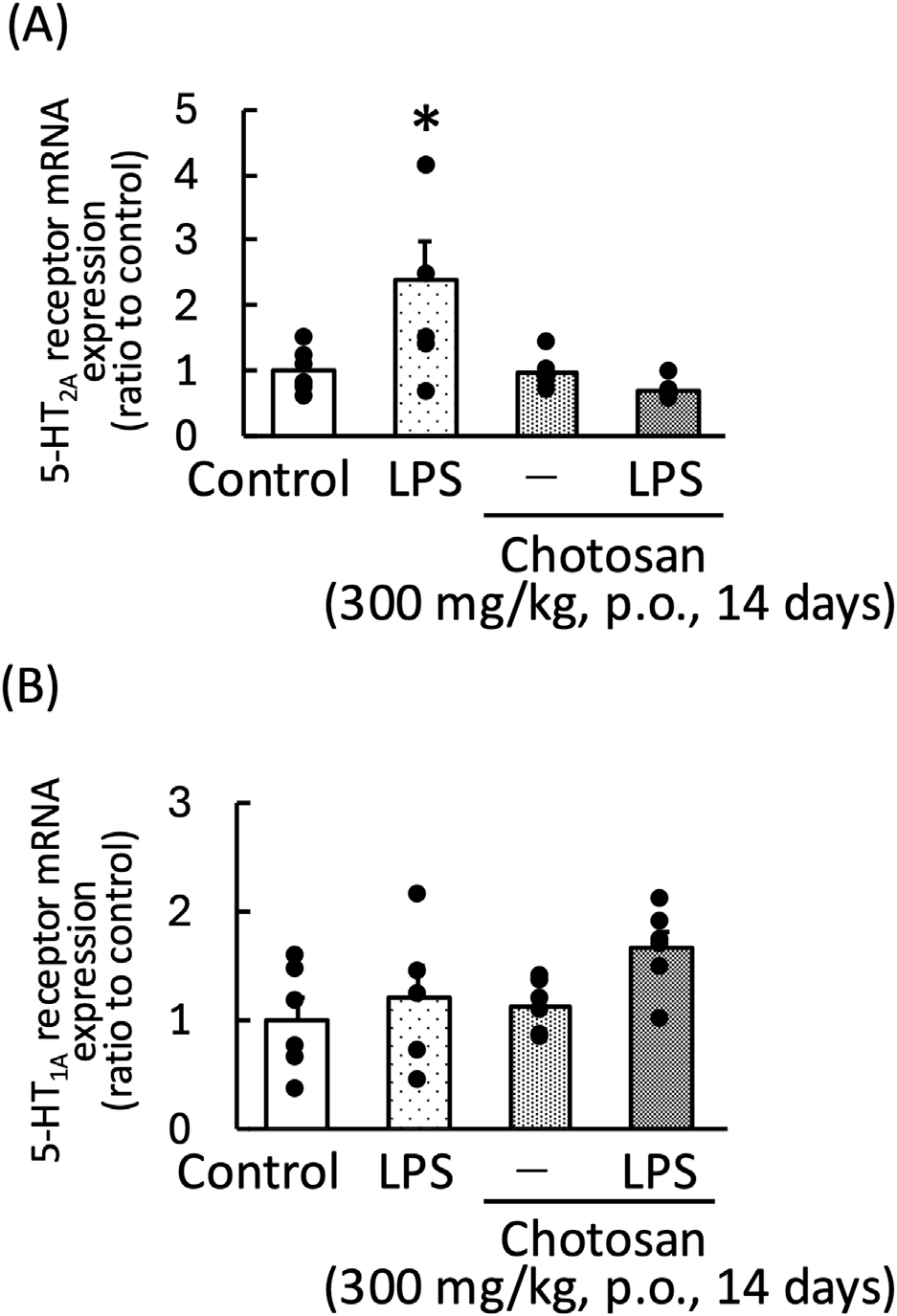
Effects of chotosan on the frontal cortex 5-HT_2A_ receptor mRNA **(A)** and hippocampal 5-HT_1A_ receptor mRNA expression **(B)** in mice. Chotosan (300 mg/kg) was administered orally for 14 days. 5-HT_2A_ receptor and 5-HT_1A_ receptor mRNA levels measured 1 day after LPS (500 μg/kg, i. p.) administration. Data are shown as mean ± standard error of the mean; n = 6 per group. Data were analyzed using two-way ANOVA, and Tukey’s test was used to compare the means of multiple groups. *p < 0.05 (vs. Control). i.p., intraperitoneal.

**FIGURE 9:**
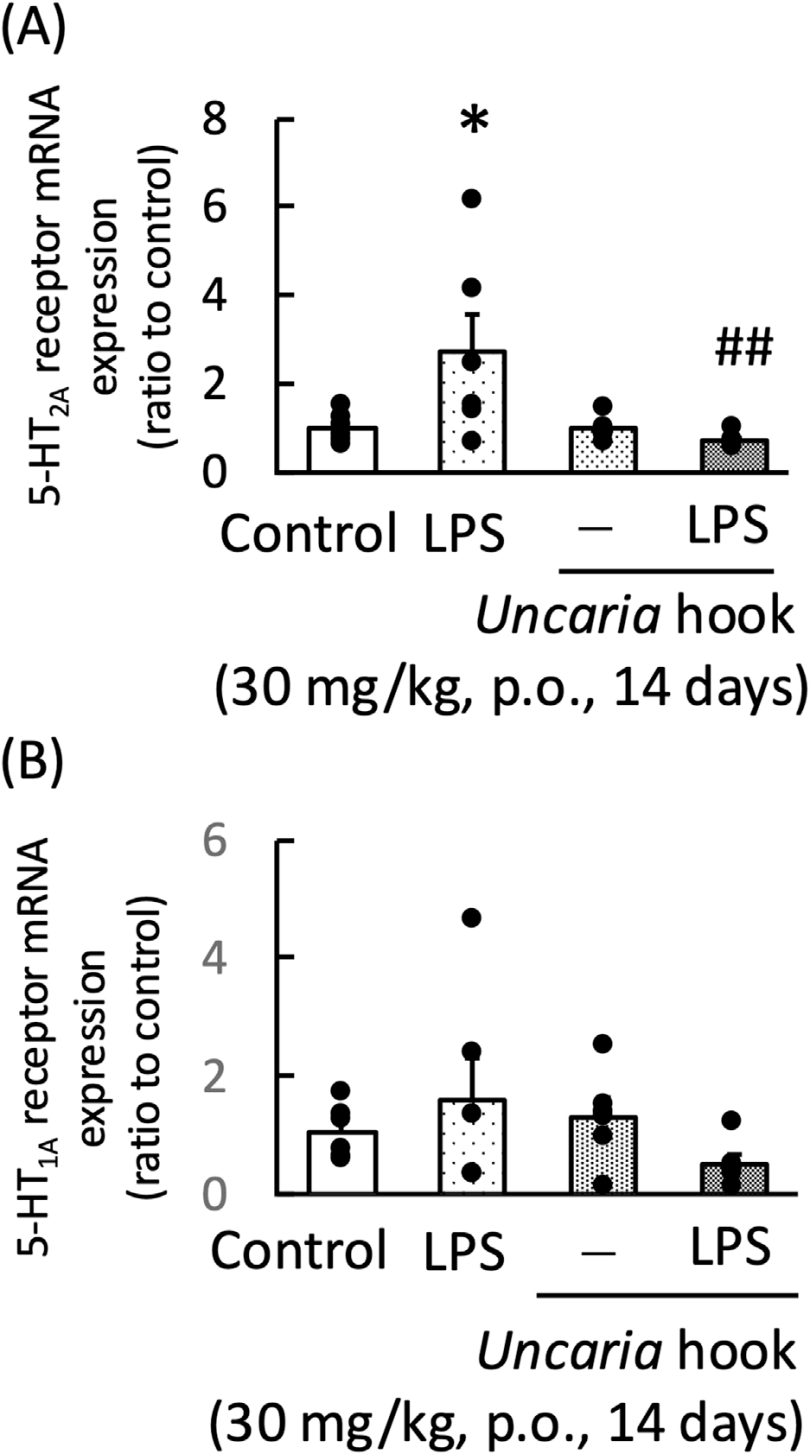
Effects of *Uncaria* hook on the frontal cortex 5-HT_2A_ receptor mRNA **(A)** and hippocampal 5-HT_1A_ receptor mRNA expression **(B)** in mice at 24 h after LPS treatment. *Uncaria* hook (30 mg/kg) was administered orally for 14 days. 5-HT_2A_ receptor and 5-HT_1A_ receptor mRNA levels measured 1 day after LPS (500 μg/kg, i. p.) administration. Data are shown as mean ± standard error of the mean; n = 6 per group. Data were analyzed using two-way ANOVA, and Tukey’s test was used to compare the means of multiple groups. *p < 0.05 (vs. Control), ##*p* < 0.01 (vs. LPS). i.p., intraperitoneal; LPS, lipopolysaccharide.

LPS treatment significantly increased the frontal cortex 5-HT_2A_ receptor mRNA expression in mice. Chotosan decreased the frontal cortex 5-HT_2A_ receptor mRNA expression in LPS-treated mice [LPS: F (1, 11) = 10.51, *p* < 0.01; chotosan: F (1, 11) = 0.35, *p* = 0.56; LPS × chotosan: F (1, 11) = 0.94, *p* = 0.35] (Figure 8A). In contrast, the hippocampal mRNA expression of the 5-HT_1A_ receptor was not affected by LPS treatment. Chotosan did not alter the hippocampal 5-HT_1A_ receptor mRNA expression in normal or LPS-treated mice [LPS: F (1, 11) = 2.92, *p* = 0.11; chotosan: F (1, 11) = 4.39, *p* = 0.06; LPS × chotosan: F (1, 11) = 0.68, *p* = 0.43] (Figure 8B). *Uncaria* hook significantly decreased the frontal cortex 5-HT_2A_ receptor mRNA expression in LPS-treated mice [LPS: F (1, 11) = 1.80, *p* = 0.21; *Uncaria* hooks: F (1, 11) = 4.29, *p* = 0.06; LPS × *Uncaria* hook: F (1, 11) = 5.11, *p* < 0.05] (Figure 9A). In contrast, the hippocampal mRNA expression of the 5-HT_1A_ receptor was not affected by treatment with LPS or *Uncaria* hook [LPS: F (1, 11) = 0.05, *p* = 0.83; *Uncaria* hook: F (1, 11) = 0.38, *p* = 0.55; LPS × *Uncaria* hook: F (1, 11) = 3.80, *p* = 0.08] (Figure 9B).

## 4 Discussion

The light–dark test employed in this study has been extensively utilized to evaluate states of anxiety and anxiolysis. Notably, we previously reported that inflammatory conditions induced by LPS administration elicited anxiety-like behaviors during the light–dark test in mice (Ushio et al., 2022). In the present study, we used the light–dark test to demonstrate that chotosan exerted anxiolytic effects in both normal and LPS-treated mice. Zhao et al. (2012) explored the effect of chotosan on emotional deficits in type 2 diabetic *db*/*db* mice, revealing these mice displayed heightened anxiety levels as assessed using the elevated plus maze. Chotosan was found to alleviate emotional deficits in these mice. Collectively, these findings suggest that chotosan possesses anxiolytic-like properties and can mitigate anxiety-like behaviors under inflammatory conditions. Regarding the active component of chotosan responsible for these effects, *Uncaria* hook, a key component of chotosan, has been previously identified (Yuzurihara et al., 2002). Our findings indicate that treatment with *Uncaria* hook significantly prolonged the duration spent by mice in light areas in the light–dark test compared with that by both vehicle- and LPS-treated mice. This observation implies that *Uncaria* hook may be the component of chotosan that contributes to its anxiolytic-like effects, particularly under inflammatory conditions. Furthermore, we acknowledge the small number of animals used in each group as a limitation of this study. As this was a screening study, the small sample size may limit the statistical power of our findings. Future studies with larger samples are necessary to confirm these results.

The 5-HT nerve system plays a pivotal role in modulating anxiety and anxiolytic effects. Among the various subtypes of 5-HT receptors, 5-HT_1A_ receptors are thought to be critically involved in the pathogenesis of mood-related disorders (Sharp and Barnes, 2020). Tandospirone, a partial agonist of the 5-HT_1A_ receptor, has anxiolytic properties (Nishitsuji et al., 2004). In this study, tandospirone induced anxiolytic-like behavior in both normal and LPS-treated mice. Notably, the anxiolytic effect of tandospirone was inhibited by WAY100635, a 5-HT_1A_ receptor antagonist. These observations suggest that agonists of the 5-HT_1A_ receptor may play a therapeutic role in alleviating anxiety-like behaviors induced by inflammation in mice. Furthermore, mice treated with chotosan spent a significantly longer time in the light zone than those treated with vehicle, and the anxiolytic effect of chotosan was attenuated by pretreatment with WAY100635. This finding implies that chotosan may induce anxiolytic-like behavior by enhancing the activity of the 5-HT_1A_ receptor. Additionally, *Uncaria* hook demonstrated anxiolytic-like effects in both normal and LPS-treated mice. This effect was also mitigated by WAY100635 treatment. A previous study reported that *Uncaria* hook exhibits partial agonistic activity toward the 5-HT_1A_ receptor (Terawaki et al., 2010). These observations collectively suggest that chotosan functions as a 5-HT_1A_ receptor agonist, potentially through its active component *Uncaria* hook, thereby exerting anxiolytic effects, especially under conditions of systemic inflammation.

It is well known that 5-HT_2A_ receptor hyperfunction is associated with neuropsychiatric disorders such as stress responses, anxiety, and depression (Shelton et al., 2009; Weisstaub et al., 2006). However, the specific role of the 5-HT_2A_ receptors in states of inflammation remains poorly understood. In this study, we investigated the mechanisms underlying LPS-induced anxiety-like behaviors, with particular emphasis on the role of 5-HT_2A_ receptor hyperactivity. Therefore, we aimed to elucidate the involvement of 5-HT_2A_ receptors in LPS-induced anxiety-like behaviors in mice. Our findings demonstrated a notable upregulation in frontal cortex 5-HT_2A_ receptor mRNA expression following LPS treatment, corroborating with the results from previous research (Couch et al., 2015). Furthermore, mice administered DOI, a 5-HT_2A_ receptor agonist, spent a shorter time in the light compartment than those subjected to normal and LPS treatments. Thus, the LPS-induced anxiety-like behaviors observed in the light–dark test might be attributed to hyperfunction of the 5-HT_2A_ receptor. Notably, we found that chotosan mitigated the elevation in 5-HT_2A_ receptor mRNA levels in LPS-treated mice, and *Uncaria* hook markedly reduced 5-HT_2A_ receptor mRNA expression in the frontal cortex of LPS-treated mice. Previous studies have documented that repeated administration of Yokukansan, which includes *Uncaria* hook, significantly reduces 5-HT_2A_ receptor protein levels in the prefrontal cortex and alleviates behaviors mediated by the 5-HT_2A_ receptor in mice (Egashira et al., 2008). Consequently, the anxiolytic-like effects observed with chotosan and *Uncaria* hooks under inflammatory conditions may be attributed to the suppression of 5-HT_2A_ receptor hyperfunction. Numerous studies have demonstrated a possible interaction between 5-HT_2A_ and 5-HT_1A_ receptors in rats and mice (Darmani et al., 1990; Dursun and Handley, 1993; Kitamura et al., 2007). Our research and previous studies have indicated that 8-OH-DPAT, a 5-HT_1A_ receptor full agonist, inhibits DOI-induced wet-dog shake behavior in rats (Darmani et al., 1990; Kitamura et al., 2007). These results support the hypothesis that 5-HT_1A_ receptors may exert an inhibitory effect on the activation of 5-HT_2A_ receptors. Furthermore, in this study, LPS treatment did not significantly modify the hippocampal 5-HT_1A_ receptor mRNA expression in mice. Additionally, *Uncaria* hook exhibits partial agonistic effects on 5-HT_1A_ receptors (Terawaki et al., 2010). These findings suggest that the anxiolytic effects of *Uncaria* hook may involve suppressive modulation of 5-HT_2A_ receptor function via the activation of 5-HT_1A_ receptors. The model of inflammatory conditions used in this study is particularly intriguing from the perspective of understanding the interplay between 5-HT_2A_ and 5-HT_1A_ receptor functions or the action of 5-HT_1A_ receptor agonists. These results contribute to a growing body of evidence suggesting that 5-HT_1A_ receptor agonists play an important role in mitigating inflammation-induced anxiety-like behaviors in mice.

We demonstrated that chotosan alleviated anxiogenic-like behavior in mice under LPS-induced inflammatory conditions, as assessed using the light–dark test. Moreover, we found that *Uncaria* hook, the key component of botanical drug chotosan, effectively suppresses anxiety-like behavior induced by LPS. The anxiety-like behavior elicited by LPS treatment appears to be associated with the hyperactivity of the 5-HT_2A_ receptor. Additionally, our findings indicate that chotosan alleviates the behavioral consequences of LPS-induced 5-HT_2A_ receptor hyperfunction. This effect is potentially mediated by its agonistic activity on the 5-HT_1A_ receptors. These results indicate the therapeutic potential of chotosan and its component (*Uncaria* hook) in ameliorating anxiety-like behavior, particularly under inflammatory conditions.

## Data Availability

The raw data supporting the conclusions of this article will be made available by the authors, without undue reservation.

## Abbreviations

LPS, lipopolysaccharide; 5-HT, serotonin; JP, Japanese Pharmacopoeia; N.D., not detected.
