# Supplementary material for: Ameliorating effect of chotosan and its active component, Uncaria hook, on lipopolysaccharide-induced anxiety-like behavior in mice
Source: Front Pharmacol. 2024 Sep 4;15:1471602. doi: 10.3389/fphar.2024.1471602 (PMC11408319; doi:10.3389/fphar.2024.1471602)
Supplement: Supplementary file 1 [file DataSheet1.docx]

Ameliorating Effect of Chotosan and Uncaria Hook on Lipopolysaccharide-Induced Anxiety-Like Behavior in Mice

## Supplementary Figures


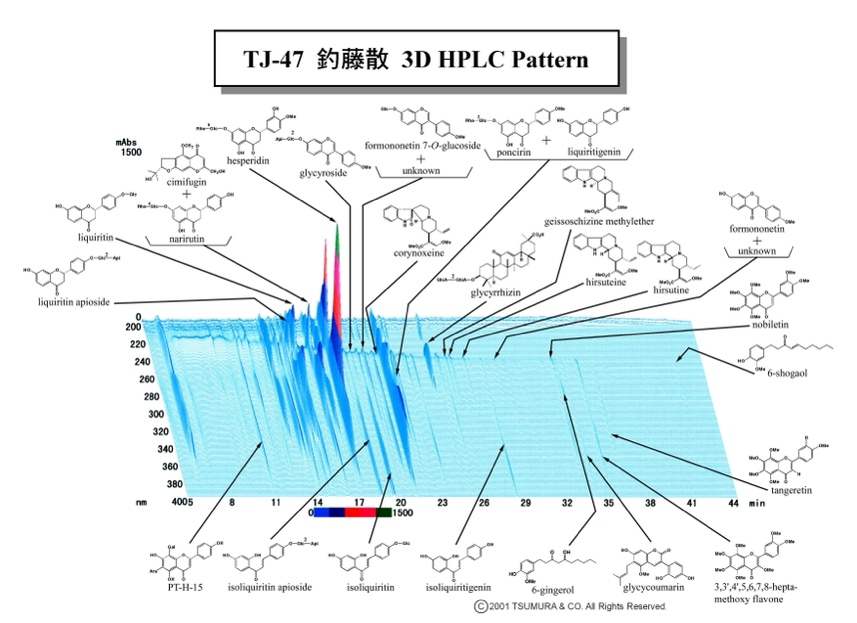


**Supplementary Figure 1.** Three-dimensional HPLC profile of chitosan


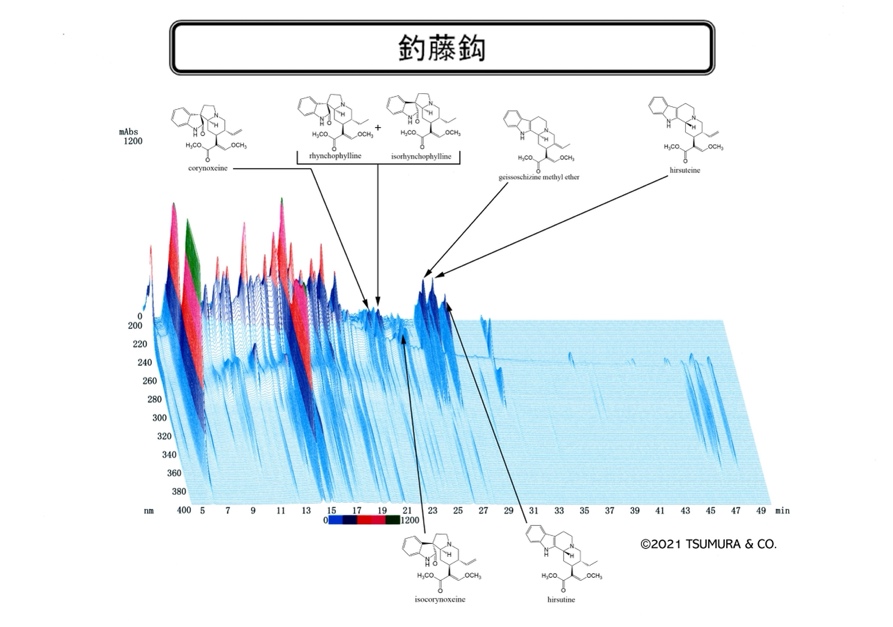


**Supplementary Figure 2.** Three-dimensional HPLC profile of Uncaria hook.
